# Foreword

**DOI:** 10.6028/jres.097.001

**Published:** 1992

**Authors:** W. C. Martin

**Affiliations:** Group Leader, Atomic Spectroscopy, Atomic Physics Division, National Institute of Standards and Technology

Accurate measurements of wavelengths in astrophysical spectra are essential for reliable line identifications in the more complex spectra and for determinations of line-of-sight velocities of the light-emitting or absorbing astronomical sources. Wavelength calibration of the highly successful (and still active) International Ultraviolet Explorer (IUE) satellite, launched in 1978, is accomplished by use of an on-board platinum hollow-cathode lamp as a source of known wavelengths against which the astrophysical spectra are measured. During the 1970s a group led by W. G, Fastie of the Johns Hopkins University developed such a platinum lamp that emitted a line-rich, bright spectrum in the 1150–3100 Å region and met all other requirements for the IUE application. The successful IUE performance led to the adoption of a platinum lamp as the on-board wavelength calibration source for the Goddard High Resolution Spectrograph (GHRS) of the Hubble Space Telescope.

The spectroscopic resolution of the GHRS in its echelle mode is about 90,000, which is almost 10 times the highest resolution of the IUE spectrometer. In 1982 the NBS (now NIST) Atomic Spectroscopy Group reviewed the literature from which the laboratory wavelengths being used for the IUE calibrations were taken and concluded that the accuracy of these wavelengths was unlikely to meet the GHRS design goals. Preliminary measurements by J. Reader and N. Acquista in 1983 showed that the relevant earlier wavelength determinations indeed had errors larger than those tolerable for the GHRS application by factors of 10 or more. During the next few years, with partial support from NASA, Reader and his coworkers used the NIST 10.7 m normal-incidence vacuum spectrograph to accurately measure some 3000 lines emitted by a platinum/neon hollow cathode lamp in the range 1032–4100 Å. These extensive measurements were consistent with new longer-wavelength determinations carried out by R. Engleman at the Los Alamos National Laboratory, In February 1986 the NIST measurements were communicated to appropriate astronomers for use in ground-based testing and calibration programs for the GHRS, and in 1990 the NIST group published the new wavelengths for about 3000 lines in the Supplement Series of the Astrophysical Journal.

These NIST measurements of the platinum/neon wavelengths are now being used for calibration of both the GHRS and the Faint Object Spectrograph of the Hubble Space Telescope, as well as for calibrating other satellite- and rocket-borne ultraviolet spectrometers. The use of a platinum/neon lamp as a source of reference wavelengths in high-resolution laboratory ultraviolet spectroscopy is also increasing. The new wavelength data for platinum have allowed extensions of the energy-level analyses of the spectra of both neutral and singly ionized platinum. These analyses, together with the accurate platinum wavelengths, will be essential data for the interpretation of recent GHRS spectra of stars having atmospheres with surprisingly high abundances of certain heavy elements, including platinum.

The full report on the NIST measurements in the form of a complete and detailed atlas of the platinum/neon spectrum presented in this special issue of the Journal of Research of NIST will be highly useful to a wide range of scientists. The photoelectric scans used to record the spectrum for the atlas have yielded relative intensities for some 5600 lines. The atlas also incorporates new line classifications from the above-mentioned major extensions of the Pt I and Pt II analyses, which were carried out by J. Blaise and J.-F. Wyart of the Laboratoire Aimé Cotton, Orsay, France; it is thus very appropriate that the other papers in this issue comprise tables of the Pt I energy levels and a detailed report on the Pt II levels. The careful, accurate, and complete measurements and analysis of the platinum spectra as carried out by physicists at NIST, Los Alamos, and Orsay will be appreciated by all users of these data for years to come.

